# Absolute CD4^+^ T cell count overstate immune recovery assessed by CD4^+^/CD8^+^ ratio in HIV-infected patients on treatment

**DOI:** 10.1371/journal.pone.0205777

**Published:** 2018-10-22

**Authors:** Yusnelkis Milanés-Guisado, Alicia Gutiérrez-Valencia, María Trujillo-Rodríguez, Nuria Espinosa, Pompeyo Viciana, Luis Fernando López-Cortés

**Affiliations:** Enfermedades Infecciosas, Microbiología Clínica y Medicina Preventiva. Instituto de Biomedicina de Sevilla/Hospital Universitario Virgen del Rocío/CSIC/Universidad de Sevilla. Seville, Spain; University of Pittsburgh Centre for Vaccine Research, UNITED STATES

## Abstract

**Objectives:**

To analyse the correlation and concordance between aCD4, CD4%, CD4/CD8, their intra-patient variability, and to compare the immune recovery (IR) rates based on the three parameters in HIV-infected patients after starting antiretroviral therapy.

**Methods:**

From a prospectively followed cohort, patients who maintained HIV-RNA suppression in ≥95% of the determinations throughout the follow-up were selected. IR was defined as aCD4 >650/μl, CD4% ≥38% or CD4/CD8 ≥1.

**Results:**

A total of 1164 patients with a median follow-up of 5 years were analysed. The increases in aCD4, CD4% and CD4/CD8 were highest during the first year and considerably lower thereafter regardless of baseline aCD4. The annual increases in aCD4 showed poor correlations with those of CD4% (r = 0.143–0.250) and CD4/CD8 (r = 0.101–0.192) but were high between CD4% and CD4/CD8 (r = 0.765–0.844; p<0.001). The median intra-annual coefficients of variation for aCD4, CD4/CD8 and CD4% were 12.5, 8.5 and 6.6, respectively. After five years, 66.7%, 41.6% and 42.1% of the patients reached aCD4 >650/μl, CD4% ≥38%, and CD4/CD8 ≥1, respectively, while only 31% achieved both aCD4 and CD4/CD8 target values.

**Conclusions:**

The increases in aCD4 poorly correlate with those of CD4% and CD4/CD8. IR rates based on aCD4 significantly overstate those obtained by CD4% and CD4/CD8. CD4% and CD4/CD8 are more stable markers than aCD4 and should be taken into account to monitor the IR after treatment initiation.

## Introduction

Conventionally, absolute CD4^+^ T lymphocyte count (aCD4) has been used both to guide clinical management of HIV-1 infection and to quantify the magnitude of immune restoration (IR) after starting antiretroviral treatment (ART). However, aCD4 shows high intra-patient variations which depend, inter alia, on the physiological variations of the total white blood cell counts and the derived lymphocyte subset values as well as tests imprecisions. By comparison, in untreated HIV-infected patients both the CD4^+^ percentage (CD4%) and the absolute CD4/CD8 ratio (CD4/CD8) are less subject to variation on repeated measurements, while their prognostic value is similar to aCD4 [1–7]. Furthermore, a low CD4/CD8 is associated with a higher risk of non-AIDS-related morbi-mortality, including incidence of non-AIDS malignancies, among ART-treated HIV-infected patients despite long-term viral suppression [8–13].

Although it is frequent to observe significant increases of aCD4 after starting ART that does not go hand in hand with changes in CD4% or CD4/CD8 ratio, there are very few studies in which IR has been evaluated based on the latter parameters [14,15]. Only one study has assessed the variability of CD4% in virologically-suppressed HIV-infected adults [7], while there is no data on the variability of CD4/CD8 in patients with ART-induced undetectable viremia. Thus, our aims were to analyze the correlation and concordance between aCD4, CD4%, CD4/CD8 and to compare the IR rates based on the three parameters. We have also assessed their intra-patient variability in virologically-suppressed subjects to evaluate whether the use of CD4% and/or CD4/CD8 are less fluctuating markers of IR than aCD4. Additionally, we propose a definition of immunologic non-responders based on CD4/CD8.

## Material and methods

### Study population

This is a retrospective study of a prospective cohort in which all treatment-naïve, HIV-infected adults (aged ≥18 years) who started ART between January 2000 and March 2016 at the Infectious Diseases Department of the Virgen del Rocío University Hospital (Seville), a public tertiary referral center which also provides specialized attention to a population of 558000 inhabitants in southern Spain.

Unlike other studies on IR [16–20], the present cohort study is limited to patients with sustained virological control to evaluate the immune systems’ maximum capacity to restore. So, patients were included if they: i) had a baseline count of aCD4, CD4% and CD4/CD8 collected ≤14 days prior to start ART; ii) received continuous ART for at least 1 year, iii) achieved an HIV-RNA <50 copies/ml during the first 24 weeks, and iv) maintained an undetectable viral load in ≥95% of the determinations throughout all the follow-up in order not to exclude subjects with occasional blips, defined as a positive viral load preceded and followed up by an undetectable viral load.

Patients with incomplete data, less than one year of follow-up, malignancies requiring chemotherapy, a diagnosis of cirrhosis with portal hypertension or hepatic encephalopathy concomitant to or within a year of starting ART, and patients with a baseline CD4/CD8 ≥1 were excluded from the analysis. By contrast, patients with aCD4 ≥650/μl were not excluded if they showed a CD4/CD8 <1, since among 222 patients attended for the first time at our facility with aCD4 ≥650/μl, only 29.7% of them had a CD4/CD8 ≥1 (M, 1.22; IQR, 1.10–1.56). The remainder 156 patients (70.3%) had a median CD4/CD8 of 0.66 (IQR, 0.51–0.81). Patients were treated according to the prevailing HIV/AIDS treatment guidelines at each time and to the best medical judgment of their responsible physicians.

### Follow-up, data collection and endpoints

Patient`s clinical assessment was performed at baseline, after a month on ART, and every three to six months thereafter, including biochemical profiles, hematologic counts, standard flow cytometry counts of aCD4, CD4% and CD4/CD8, and plasma HIV-RNA levels measured by polymerase chain reaction with lower detection limits from 20 to 50 copies/ml depending on the available technique at any time. For this study, data on aCD4, CD4%, and CD4/CD8 were collected at baseline and subsequently on an annual basis. To be included in each annual analysis, the patients had to have an aCD4, CD4% and CD4/CD8 determination obtained within 2 months of the annual anniversary date of ART start. The determination closest to the annual anniversary date was used for analysis.

Patients were analyzed until the last available determination of aCD4, CD4% and CD4/CD8 and were censored in case of the following events, whichever occurred first: virological failure, defined as the first of two consecutive plasma viral loads >200 copies/ml separated at least by one week according to the DHHS Panel on Antiretroviral Guidelines for Adults and Adolescents recommendation [21], treatment interruption, loss to follow-up, a diagnosis of cirrhosis with portal hypertension or hepatic encephalopathy, malignancy, and death. Data were retrieved from routinely collected, comprehensive electronic medical records covering all HIV-infected patients receiving care at our center who provided written informed consent at the beginning of the follow-up. The study was approved by the Ethics Committee of Virgen del Rocío University Hospital.

We firstly analyzed the correlations and concordance between all the simultaneous aCD4, CD4% and CD4/CD8 determinations from the analyzed patients. Afterwards, we analyzed the IR rates, defined as a confirmed aCD4 ≥650/μl, a CD4% ≥38% or a CD4/CD8 ≥1, according to the aCD4 strata at baseline. A CD4/CD8 ≥1 was selected as it has been associated to lower mortality in the general elderly population and also to lower levels of immune activation in subjects treated for HIV infection [22–24]. The cut-off points of aCD4 ≥650/μl, and CD4% ≥38% were selected based on our results as the values with the higher intraclass correlation coefficients with a CD4/CD8 ≥1 (see below). Moreover, we assessed the rate of extensive immune restoration (eIR), defined as the achievement of aCD4 ≥650/μl plus CD4/CD8 ≥1 [15]. Finally, we evaluated the annual intra-patient variability of CD4% and CD4/CD8 compared with that of aCD4 in stable, virologically-suppressed subjects.

### Statistical analysis

Quantitative variables were expressed as median, interquartile range (IQR) or range, and categorical variables as number and percentage. The correlations between aCD4, CD4%, and CD4/CD8 were assessed by Pearson correlation coefficients. The cut-off points of ≤200, 201–350, 351–500, and >500/μl have been conventionally used to describe the immunology situation of these patients. On the basis of all the available determinations, we calculated the corresponding CD4% and CD4/CD8 with the higher intraclass correlation coefficients and Lin concordance correlation coefficients (r_c_) [25]. The χ^2^ test was used to compare categorical variables, while the Mann–Whitney U test and the Kruskal–Wallis test were used for quantitative variables. In addition to absolute increases, the percentage changes of the aCD4, CD4% and CD4/CD8 were calculated to take into account the baseline values using the following formula: (value at a specific time-point—baseline value)/basal value *100. The time to IR and eIR was estimated using Kaplan–Meier curves and Cox regression models. The differences between the curves were assessed using the log-rank test. Covariates in the final model included the period of ART introduction (2000–2005, 2006–2010, ≥2011), age (per 10-year increment), gender, mode of HIV transmission, HCV and HBV co-infection, and aCD4, CD4% or CD4/CD8 at ART initiation. Continuous variables were log-transformed when necessary to satisfy model assumptions. Coefficients of variation (CV) were used to compare the annual intra-patient variability of aCD4, CD4% and CD4/CD8 from the third year onwards. Statistical analyses were performed using R commander, p-values <0.05 were considered significant.

## Results

### Study population

From January 2000 to March 2016, 2,484 treatment-naive HIV-infected adults initiated ART at our department. Of these, 1,320 were excluded from the analysis due to any of the exclusion criteria above mentioned as depicted (S1 Fig). Thus, 1,164 subjects with a median follow-up of 5 years (IQR, 3–8; range, 1–15) were analyzed. The main characteristics of the study population at baseline are shown in Table 1. Sixty-nine (5.9%) patients with a basal aCD4 ≥650/μl and CD4/CD8 <1 (median, 0.59; IQR, 0.46–0.71; range, 0.23–0.95) were also included.

**Table 1:** Patients’ characteristics at baseline.

	n = 1164
**Male sex, no. (%)**	1013 (87)
**Age (years)**	36 (29–43) [18–73]
**Caucasian, n (%)**	1091 (93.7)
**Risk factor for HIV, no. (%)**	
Previous iv drug use	145 (12.5)
Homosexual	744 (63.9)
Other	275 (23.6)
**HIV-RNA, log**_**10**_ **copies/ml**	4.80 (4.35–5.20) [2.04–7.00]
**CD4 count/μl**	281 (157–409) [2–1276]
≤ 200, n = 380	97 (38–156)
201–350, n = 377	278 (244–309)
351–500, n = 239	411 (380–454)
501–649, n = 99	564 (528–584)
≥ 650, n = 69	735 (697–816)
**CD4%**	18.0 (12.1–24.3) [0.10–47.58]
≤ 16%, n = 477	11.0 (6–13.9)
16.1–24%, n = 385	19.9 (17.9–21.9)
24.1–32%, n = 222	27.5 (25.5–29.4)
32.1–37.9%, n = 62	34.1 (32.8–35.1)
≥ 38%, n = 18	39.6 (38.0–42.7)
**CD4/CD8 ratio**	0.31 (0.18–0.46) [0.01–0.99]
< 0.30, n = 560	0.17 (0.10–0.23)
0.30–0.50, n = 368	0.38 (0.34–0.43)
0.51–0.79, n = 205	0.61 (0.56–0.68)
0.80–<1, n = 31	0.90 (0.83–0.94)
**HCV-RNA +, no. (%)**	177 (15.2)
**HBsAg+, no. (%)**	54 (4.6)
**AIDS events, no. (%)**	184 (15.8)

Quantitative variables expressed as median, (IQR) and [range].

### Correlations and concordances between aCD4, CD4% and CD4/CD8

Based on the analysis of 21,743 simultaneous determinations of aCD4, CD4% and CD4/CD8 throughout the follow-up of the analyzed patients, we found positive correlations between aCD4 and CD4% (r = 0.620, p <0.001) and between aCD4 and CD4/CD8 ratio (r = 0.597, p <0.001), but the strongest correlations were between CD4% and CD4/CD8 ratio both in the whole sample (r = 0.903; p <0.001) and in the different aCD4 strata (r = 0.851–0.917; p <0.001) (S2 Fig and S1 Table).

Besides that, the corresponding values that showed highest concordance with the aCD4 cut-off points of ≤200, 201–350, 351–500, 501–650, and >650/μl were <0.30, 0.30–0.50, >0.50–0.79, 0.80–0.99 and ≥1, respectively, for CD4/CD8 (Cronbach`s α = 0.764, p <0.001; r_c_ = 0.587, CI_95_, 0.578–0.595), and ≤16, 16.1–24, 24.1–32, 32.1–37.9, and ≥38%, respectively, for CD4% (Cronbach`s α = 0.871, p <0.001; r_c_ = 0.691, CI_95_, 0.684–0.698). Once again, the level of concordance between the selected CD4% and CD4/CD8 cut-off points was the highest (Cronbach`s α = 0.937, p <0.001; r_c_ = 0.884, CI_95_, 0.875–0.881).

### Dynamics of aCD4, CD4%, and CD4/CD8 and correlations between them

The greatest increases in aCD4, CD4% and CD4/CD8 occurred during the first year in all the aCD4 strata. The increase in aCD4 was similar in the strata of 200–350 and 350–500 (pooled data: M, 216/μl; IQR, 131–325) followed some way behind by those who initiated ART with ≤200/μl (197; IQR, 127–292), and, finally, those in the higher aCD4 stratum (108; IQR, -33–316) (p <0.001). The lower increase in aCD4 in patients with aCD4 ≥650/μl is not surprising as their values were already in a normal to near-normal range; however, CD4% and CD4/CD8 continued to increase in the same proportion as in the other groups.

On the other hand, during the first year CD4% increased similarly regardless of the aCD4 strata (pooled data, 8.7%; IQR, 5.1–12.3). Furthermore, the increases in CD4/CD8 also were similar in the different aCD4 strata except in those with ≤200/μl at baseline (0.31; IQR, 0.18–0.46 vs. 0.19; IQR, 0.10–0.31; p <0.001). This lower increase seems justified by the concomitant increase in absolute CD8^+^ T cells (aCD8) (222/μl; IQR, 16–554) which was positively correlated with the gain in aCD4 (r = 0.357; p <0.001). By contrast, there was a significant aCD8 decrease (M, -174; IQR, -440–37) inversely proportional to the basal values (r = -0.131; p = 0.035) in patients with aCD4 ≥350/μl at baseline. This decrease was much smaller in patients with aCD4 values between 201 and 350/μl (-20; IQR, -243–192). In fact, if we normalize the increase of CD4/CD8 by the increase of aCD8 during the first year, the differences disappear (Table 2).

**Table 2:** Annual absolute increases in CD4 cell counts (aCD4), CD4 percentage (CD4%), CD8+ T cell counts, and CD4/CD8 ratio (CD4/CD8).

Groups		Year 1	Year 2	Year 3	Year 4	Year 5	Year 6	Year 7	Year 8	Year 9	Year 10
		n = 372	n = 331	n = 300	n = 275	n = 241	n = 200	n = 165	n = 149	n = 111	n = 104
**≤200**	**Δ aCD4**	197 (127–292)*	70 (7–128)	50 (-23–115)	29 (-27–114)	22 (-42–102)	19 (-51–96)	33 (-42–109)	16 (-61–86)	23 (-45–126)	-5 (-82–69)
	**Δ CD4%**	8.3 (5.0–11.9)	3.1 (1.0–5.2)	2.2 (-0.1–4.8)	2.1 (-0.1–4.3)	1.5 (-0.8–3.6)	1.04 (-1.07–2.98)	0.80 (-1.2–3.60)	0.50 (-1.8–2.25)	1.55 (-0.8–3.79)	0.60 (-1.50–2.36)
	**Δ CD8**	222 (16–554)	-42 (-181–143)	-20 (-198–133)	-29 (-175–115)	-23 (-169–97)	-17 (-174–120)	-4 (-101–143)	-1 (-127–151)	-3 (-146–128)	-35 (-182–131)
	**Δ CD4/CD8**	0.19 (0.10–0.31)^§^	0.08 (0.03–0.16)	0.06 (0.01–0.14)	0.06 (-0.01–0.12)	0.06 (-0.01–0.11)	0.04 (-0.02–0.11)	0.03 (-0.03–0.12)	0.02 (-0.05–0.12)	0.03 (-0.01–0.09)	0.03 (-0.06–0.09)
		n = 370	n = 333	n = 299	n = 260	n = 222	n = 176	n = 130	n = 109	n = 62	n = 54
**201–350**	**Δ aCD4**	246 (171–344)*	50 (-30–151)	49 (-41–139)	27 (-58–130)	23 (-74–128)	46 (-75–138)	25 (-95–105)	6 (-96–110)	68 (-85–197)	-18 (-159–123)
	**Δ CD4%**	9.4 (6.1–12.9)	2.7 (0.0–5.6)	1.6 (-0.9–4.1)	1.6 (-1.1–4.1)	0.8 (-1.8–3.4)	0.8 (-1.9–2.9)	0.7 (-1.54–3.24)	0.59 (-2.04–2.90)	0.79 (-1.8–3.74)	0.30 (-2.5–2.9)
	**Δ CD8**	-20 (-243–192)	-48 (-202–106)	-5 (-152–152)	-28 (-168–125)	-18 (-133–151)	2 (-108–146)	-37 (-146–111)	13 (-134–113)	44 (-60–124)	-53 (-144–92)
	**Δ CD4/CD8**	0.30 (0.16–0.44)^§^	0.11 (0.02–0.21	0.06 (-0.12–0.17)	0.05 (-0.02–0.16)	0.04 (-0.07–0.13)	0.03 (-0.05–0.10)	0.02 (-0.05–0.14)	0.03 (-0.07–0.12)	0.03 (-0.06–0.13)	0.02 (-0.06–0.11)
		n = 236	n = 215	n = 177	n = 144	n = 101	n = 63	n = 34	n = 25	n = 12	n = 11
**351–500**	**Δ aCD4**	233 (135–351)*	60 (-48–171)	44 (-53–163)	8 (-91–139)	12 (-80–99)	5 (-54–92)	19 (-216–135)	36 (-88–171)	30 (-114–52)	-73 (-121–76)
	**Δ CD4%**	8.6 (4.7–12.3)	2.2 (-0.1–4.8)	1.2 (-0.9–4.2)	0.5 (-2.0–3.7)	0.9 (-1.6–3.2)	0.7 (-1.8–2.9)	0.8 (-0.69–3.56)	0.1 (-0.16–0.60)	2.3 (0.11–3.87)	1.0 (-0.97–3.35)
	**Δ CD8**	-123 (-347–111)	-70 (-197–106)	-30 (-160–189)	-16 (-139–108)	-13 (-162–107)	16 (-116–113)	-26 (-187–218)	65 (-124–62)	-64 (-124–62)	-61 (-252–39)
	**Δ CD4/CD8**	0.30 (0.18–0.47)^§^	0.10 (0.00–0.17)	0.06 (-0.01–0.17)	0.05 (-0.05–0.15)	0.03 (-0.04–0.16)	0.01 (-0.07–0.09)	0.01 (-0.09–0.11)	0.02 (-0.09–0.07)	0.06 (-0.07–0.15)	0.08 (-0.12–0.19)
		n = 98	n = 77	n = 51	n = 33	n = 26	n = 17				
**501–650**	**Δ aCD4**	241 (112–391)*	43 (-89–171)	15 (-116–123)	85 (-19–242)	4 (-139–191)	-66 (-112–206)				
	**Δ CD4%**	8.5 (4.43–13.7)	1.8 (-1.4–4.2)	1.3 (-1.4–4.3)	0.9 (-1.9–4.1)	-0.5 (-3.5–2.9)	2.3 (-3.0–5.38)				
	**Δ CD8**	-226 (-498–38)	-57 (-202–144)	-39 (-231–123)	-14 (-187–256)	8 (-159–286)	-2 (-209–118)				
	**Δ CD4/CD8**	0.37 (0.22–0.52)^§^	0.08 (-0.02–0.18)	0.06 (-0.05–0.17)	0.06 (-0.04–0.15)	0.01 (-0.10–0.13)	0.07 (-0.10–0.26)				
		n = 69	n = 49	n = 33	n = 16	n = 14					
**>650**	**Δ aCD4**	108 (-33–316)*	-6 (-133–229)	21 (-67–134)	52 (-111–296)	6 (-127–166)					
	**Δ CD4%**	6.8 (4.2–10.9)	2.0 (-0.9–5.5)	1.8 (-0.4–5.2)	0.3 (-4.1–1.6)	1.2 (-2.1–5.2)					
	**Δ CD8**	-393 (-611–107)	-111 (-276–173)	-63 (-265–73)	6 (-163–227)	-33 (-78–101)					
	**Δ CD4/CD8**	0.32 (0.22–0.48)^§^	0.08 (-0.05–0.19)	0.09 (0.10–0.19)	0.07 (-0.09–0.10)	0.03 (-0.04–0.16)					

Median, (IQR).

* and § p<0.001.

All other comparison within each year, p<0.200. Data for the years 6 to 10 have been removed in groups with less than ten subjects per year.

From the second year onward, the increases in the three markers were smaller every year and without statistical differences among the different basal aCD4 strata. The aCD4 increased progressively until reaching a plateau at the fifth year among subjects who started ART with >500/μl, and the ninth year for subjects starting ART below this level. By contrast, the CD4/CD8 continued to increase until the tenth year at least in patients who initiated ART with aCD4 ≤500/μl; for those with higher values the sample size was too small to obtain conclusions beyond the fifth year (Table 2 and S3 Fig). The three parameters tended to increase over time in most patients, but low correlations were observed between the year to year increases of aCD4 and CD4% (r = 0.143–0.250; p <0.001), and between aCD4 and CD4/CD8 (r = 0.101–0.192; p <0.001); however, the correlations were much better between the increases of CD4% and CD4/CD8 (r = 0.765–0.844; p <0.001). Regarding the aCD8, there were moderate decreases, without significant differences among the aCD4 strata from year to year (p = 0.100–0.983), with median values which tended to stabilize around 800/μl at the fourth to fifth year (Table 2 and S4 Fig).

### Cumulative probabilities of immune recovery

**Fig 1:**
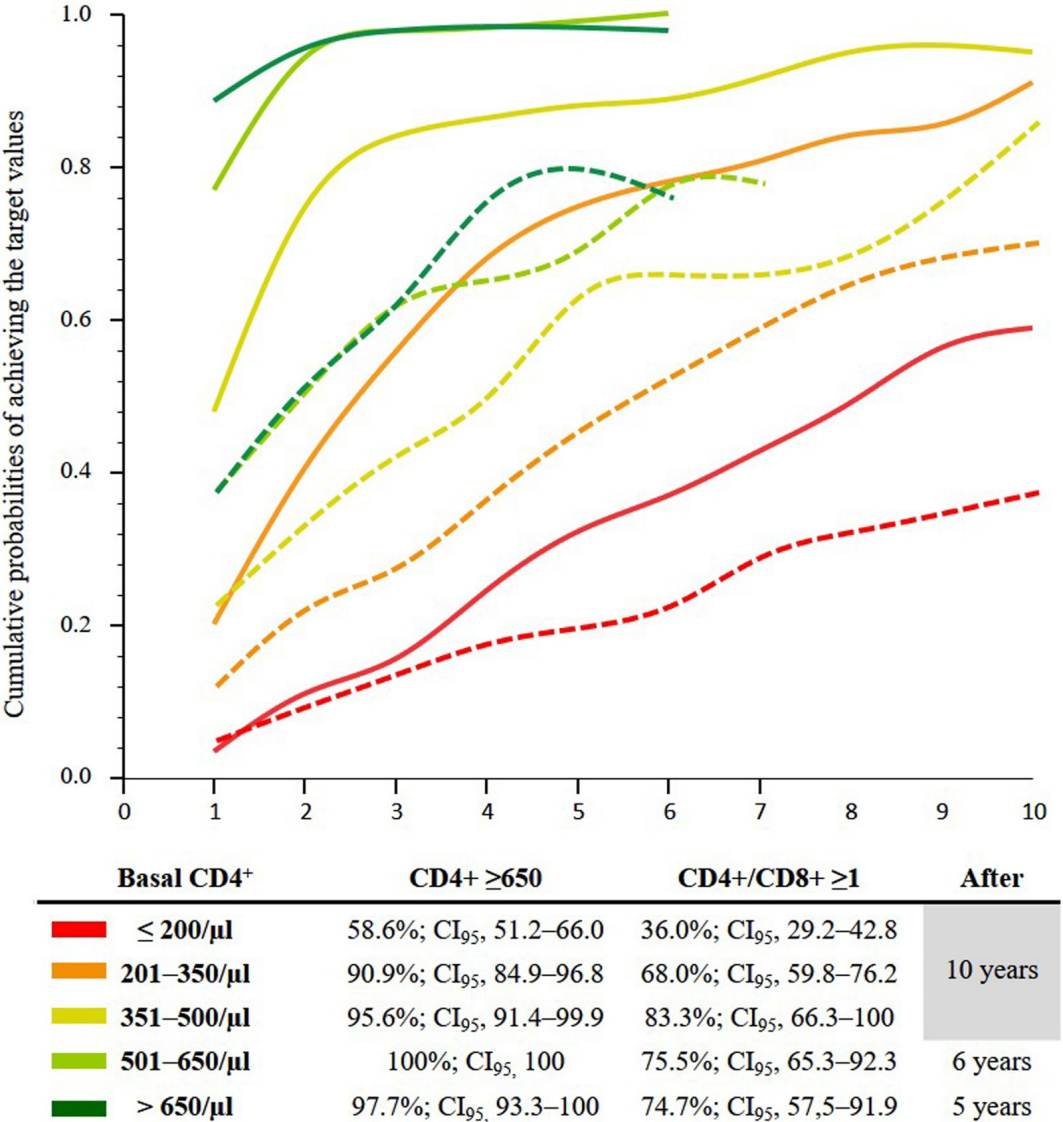
Cumulative probabilities of achieving a CD4 count ≥650/μl (continuous lines) and a CD4/CD8 ratio ≥1 (dashed lines) after starting antiretroviral therapy as function of CD4 counts at baseline. * the values for patients who started treatment with absolute CD4 between 500 and 650/μl and >650/μl were displayed up to 6 and 5 years of follow-up, respectively, as the number in these groups decreased to less than 10 patients from these points on.

After five years 31.9, 74.6, 87.9, and 98.9% of the subjects with basal aCD4 ≤200, 201–350, 351–500 and ≥500/μl, respectively, achieved an aCD4 ≥650/μl. These probabilities were much lower for attaining a CD4/CD8 ≥1 (18.9, 44.2, 61.9, and 69.4%; p <0.001), and further decreased for achieving eIR (9.3, 27.8, 51.6, and 63.4%, respectively; p <0.001) (Table 3, and Fig 1) so that the IR rates assessed by aCD4 clearly overstate that one measured by CD4% and CD4/CD8.

**Table 3:** Cumulative probabilities of achieving absolute CD4 ≥650/μl, CD4% ≥38%, a CD4/CD8 ratio ≥1, and extensive immune recovery (eIR), defined as achieving an aCD4 ≥650/μl plus a CD4/CD8 ratio ≥1, by years of follow-up expressed as percent (CI_95_).

CD4 strata	Year 1, % (CI_95_) n = 1159	Year 3, % (CI_95_) n = 883	Year 5, % (CI_95_) n = 616	Year 7, % (CI_95_) n = 344	Year 10, % (CI_95_) n = 175	p
**Absolute CD4 ≥650/μl**						
≤200	3.4 (1.6–5.2)	15.4 (11.4–19.1)	31.9 (26.5–37.3)	42.5 (36.2–48.8)	58.6 (51.2–66.0)	<0.001
201–350	19.9 (15.9–23.9)	55.6 (50.3–60.9)	74.6 (69.5–79.7)	80.6 (75.5–85.6)	90.9 (84.9–96.8)	
351–500	47.7 (41.4–54.0)	83.9 (78.9–88.9)	87.9 (83.2–92.6)	91.3 (86.5–96.0)	95.6 (91.4–99.9)	
501–650	76.8 (68.4–85.1)	94.2 (89.3–99.1)	100 (100)	**–**	**–**	
>650	88.4 (80.9–96.0)	95.4 (93.3–101.2)	97.7 (93.3–102)	**–**	**–**	
All patients	29.1 (26.5–31.7)	54.3 (51.3–57.3)	66.7 (63.7–69.7)	72.9 (69.8–76.0)	81.7 (78.4–85.0)	*
**CD4% ≥38%**						
≤200	2.4 (0.8–3.9)	8.2 (5.3–11.2)	14.4 (10.4–18.3)	22.0 (16.7–27.2)	33.1 (26.1–40.0)	<0.001
201–350	9.5 (6.6–12.5)	27.2 (23.4–32.9)	46.7 (41.0–52.5)	56.3 (49.3–62.8)	64.2 (56.2–72.4)	
351–500	19.2 (14.2–24.2)	44.5 (37.7–51.2)	59.1 (51.2–67.0)	63.3 (54.1–72.6)	75.6 (55.1–96.1)	
501–650	35.4 (25.9–44.8)	57.6 (46.3–69.0)	72.8 (58.4–87.1)	90.9 (75.6–106.2)	100 (100)	
>650	42.0 (30.4–53.7)	73.8 (61.8–85.9)	84.3 (70.9–97.7)	**–**	**–**	
All patients	13.3 (11.4–15.3)	29.6 (26.8–32.3)	41.6 (38.3–44.9)	49.0 (45.3–52.7)	57.1 (52.6–61.5)	* ɸ
**CD4/CD8 ratio ≥1**						
≤200	4.5 (2.4–6.6)	13.0 (9.5–16.6)	18.9 (14.6–23.3)	28.1 (22.4–33.7)	36.1 (29.4–42.9)	<0.001
201–350	11.4 (8.2–14.6)	26.7 (22.0–31.4)	44.2 (38.4–49.9)	57.4 (50.7–64.2)	68.0 (59.8–76.1)	
351–500	21.3 (16.1–26.5)	41.5 (34.9–48.1)	61.9 (54.1–69.7)	67.2 (58.2–76.2)	83.6 (66.9–100.3)	
501–650	36.4 (26.9–45.8)	60.2 (49.0–71.4)	67.3 (54.4–80.3)	75.5 (58.5–92.4)	–	
>650	36.2 (24.9–47.6)	60.5 (47.2–73.9)	73.7 (56.4–91.0)	–	–	
All patients	14.8 (12.7–16.8)	29.7 (27.0–32.4)	42.1 (38.8–45.4)	50.9 (47.2–54.6)	58.9 (54.5–63.3)	* ɸ
**aCD4 ≥650/μl *plus* CD4/CD8 ≥1**						
≤200	0.5 (-0.2–1.3)	5.1 (2.8–7.5)	9.3 (6.0–12.6)	15.6 (10.9–20.2)	24.4 (17.9–30.9)	<0.001
201–350	3.4 (1.6–5.3)	15.3 (11.5–19.2)	27.8 (22.6–33.3)	40.3 (33.6–46.9)	55.1 (45.6–64.6)	
351–500	15.1 (10.5–19.6)	33.9 (27.5–40.3)	51.6 (43.7–59.4)	57.5 (48.5–66.6)	–	
501–650	30.3 (21.3–39.4)	56.5 (45.1–67.8)	63.4 (50.3–76.6)	78.1 (60.5–95.6)	–	
>650	33.3 (22.2–44.5)	58.6 (45.0–72.2)	76.3 (59.2–93.4)	–	–	
All patients	8.9 (7.3–10.6)	21.3 (18.8–23.8)	31.0 (27.9–34.0)	39.0 (35.3–42.6)	47.5 (43.9–52.6)	#

*, p <0.001 for comparisons between achieving an aCD4 ≥650/μl vs CD4% ≥38% or a CD4/CD8 ratio ≥1 at each time-point.

ɸ, p = 0.573 to 0.803 for comparisons between achieving a percent of CD4 ≥ 38% versus a CD4/CD8 ratio ≥1 at each time-point. #, p <0.001 for comparisons between achieving a CD4/CD8 ratio ≥1 versus eIR at each time-point.

To analyze the factors associated with IR, in the multivariate models, after adjusting for other covariates, higher HIV-RNA at baseline, and higher aCD4, CD4% or CD4/CD8 at baseline were consistently associated with reaching an aCD4 ≥650/μl and a CD4/CD8 ≥ 1. Three multivariate analyses were performed for eIR fitted with the sole basal aCD4, CD4% or CD4/CD8 ratio. In the three models, female gender, no HCV-co-infection, as well as higher HIV-RNA, aCD4, CD4% or CD4/CD8 at baseline were associated with an increased probability of achieving the eIR. Neither age, HBV-confection, nor the period of ART introduction were consistently associated with achieving the target values (S2, S3 and S4 Tables). According to the sensitivity analysis, all statistical studies were performed excluding subjects with aCD4 ≥650 and CD4% ≥38% at baseline (n = 1,084). The factors associated with IR were the same as those found in the main analysis (data not shown).

### Intra-patient variability of aCD4, CD4%, and CD4/CD8

**Fig 2:**
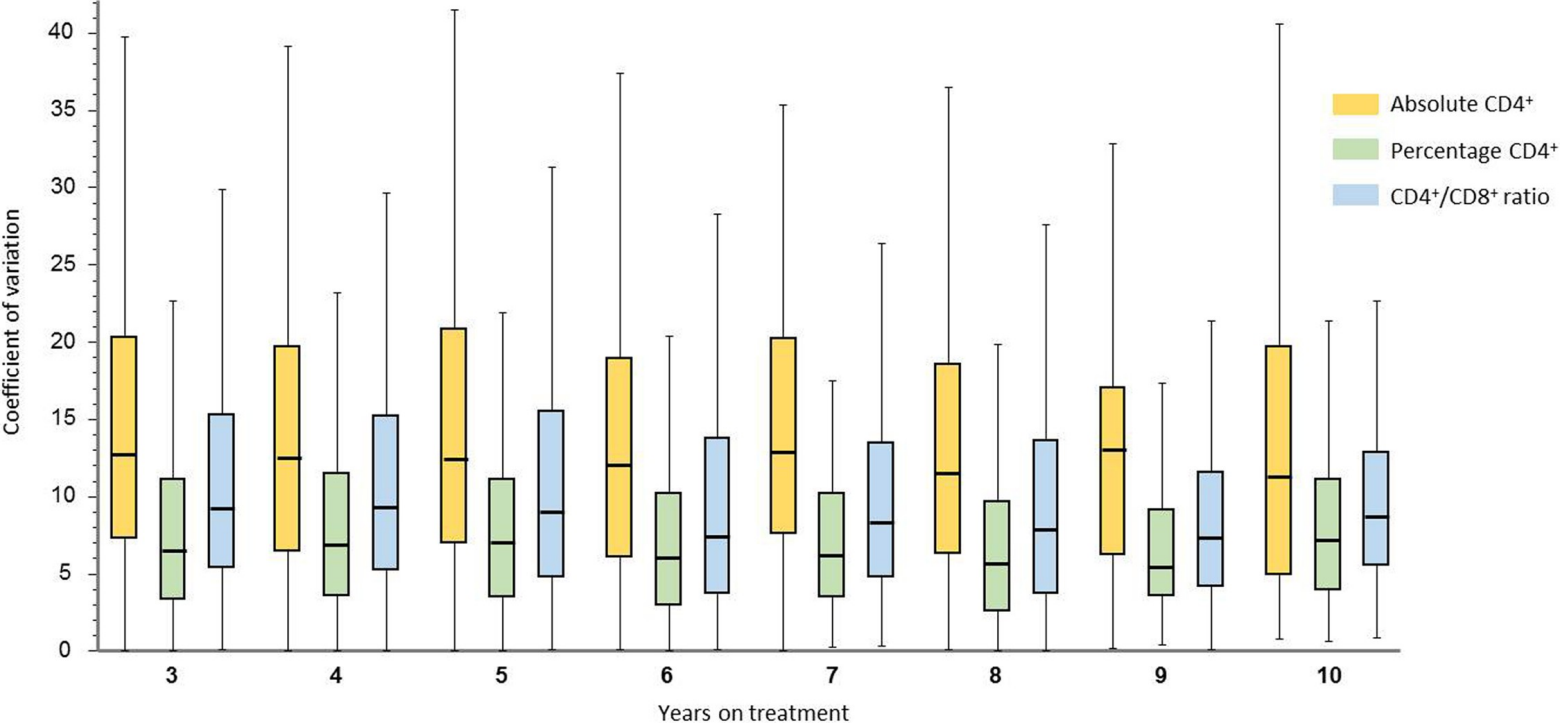
Intra-patient variability of absolute CD4 T cell counts, percent of CD4, and CD4/CD8 ratios from the third to the tenth year of sustained HIV viremia control. p <0.001 for all comparisons between absolute CD4 T cell counts and CD4/CD8, except for the tenth year, p = 0.057). p <0.001 for all comparisons between percentage of CD4 and absolute CD4 T cell counts and CD4/CD8, respectively.

As shown in Fig 2, based on 8,674 determinations from 692 patients as of the third to the tenth year, the median year to year intra-patient CV for aCD4 (M, 12.5%; IQR, 8.8–16.9) were higher than for CD4/CD8 (8.5%; IQR, 5.8–13.0) (p <0.001 for every year, except for the tenth year, p = 0.057), while the lowest intra-patient CV were observed for CD4% (6.6%; IQR, 4.2–9.8) (p <0.001 for all comparisons with aCD4 and CD4CD8, respectively).

### Immunological non-responders

Some individuals, usually referred to as immunological non-responders (INRs), experience limited recovery of aCD4 despite a prolonged undetectable viral load. Its definition lacks of consensus, but always has been based in the increase of aCD4 above different thresholds over a given time period [26]. In a post hoc analysis patients were grouped as INR or responders based on the increase in aCD4 or CD4/CD8 from basal values to the second year on treatment using the values of the different percentiles of the whole sample. We have considered as INR those patients who began ART with aCD4 <400/μl [27] and had a rise in aCD4 or CD4/CD8 lower than the percentiles 15^th^ after one and two years on ART (aCD4 ≤100 and ≤150 cells/μl; CD4/CD8 ≤0.1 and ≤0.15, respectively). Based on the aCD4 increase, only 9.4% (80/853) of the patients would be classified as INR with cumulative probabilities of achieving aCD4 >650/μl, CD4/CD8 ≥1, and eIR after five years were 2.2% (CI_95_, 0.0–6.5), 13.8% (CI_95_, 4.8–23.8), and 0%, respectively. However, based on the CD4/CD8 increase the INR percentage would rise up to 20.2% (168/831) with cumulative probabilities of achieving aCD4 >650/μl, CD4/CD8 ≥1, and eIR after five years were 38.5% (CI_95_, 30.3–47.0), 6.1% (CI_95_, 1.6–10.6) and 2.5% (CI_95_, 0.0–5.0), respectively, which again confirms the idea that IR rates assessed by aCD4 clearly overstate that one measured by CD4/CD8. Nevertheless, only 40 subjects were classified as INRs by both criteria, with a kappa coefficient of 0.176. S5 Table shows the characteristic of both populations.

## Discussion

In addition to avoiding the morbidity and mortality associated with HIV infection, one of the current objectives of ART is to achieve an IR as complete as possible. Commonly, IR is assessed by aCD4 in spite of its considerable intra-person variability between successive measurements both in patients as well as in healthy subjects [1,4]. In our cohort of treated patients we have observed similar correlations between the three T cell markers to those described by Taylor et al. in ART-naïve patients [1] and, once again, the best correlations were between CD4% and CD4/CD8, both in the overall population, as well as in the different aCD4 strata.

While several studies on aCD4 dynamics in patients with long-term sustained virological suppression below 200 copies/ml have been reported [28–31], very scarce information on CD4/CD8 restoration is available [10,30]. As previously described for aCD4 [29], the highest increases in aCD4, CD4% and CD4/CD8, both absolute and proportional changes, occurred mainly during the first year in all the aCD4 strata. From that point onwards, the increases were much smaller and, surprisingly, similar independently of the aCD4 at baseline.

There were no differences in the IR rates based on CD4% and CD4/CD8 which, furthermore, correlated well. By contrast, it is worth noting that the increases in aCD4 showed poor correlations with those of CD4% and CD4/CD8 and that the IR rates based on aCD4 significantly overstated that assessed by CD4% and CD4CD8. While two-thirds of the patients in our cohort achieved an aCD4 ≥650/μl after five years on ART, only forty percent achieved a CD4/CD8 ≥1, and less than a third achieved an optimum scenario of both aCD4 ≥650/μl plus CD4/CD8 ≥1. Even among those who initiated ART with aCD4 ≥500/μl, this aim was achieved only by two-thirds of the patients after five years of controlled viremia. The CD4/CD8 ratio, additionally, would allow identifying patients who, although they have high aCD4, maintain a persistent dysregulation of the immune system that is associated with a potentially unfavorable outcome.

In accordance with previous studies [15,32], we found that female gender, no HCV-co-infection, and higher HIV-RNA, aCD4, CD4% or CD4/CD8 at baseline were consistently associated with an increased probability of reaching the target values of IR. Thus, starting ART as early as possible offers the best opportunity for maximal immune recovery [33]. It is noted that the period of ART introduction was not associated with achieving the IR target values. This may be largely due to the fact that only patients who maintained an undetectable viral load in ≥95% of the determinations throughout all the follow-up were analyzed, regardless of the prevailing ART used in each period.

So far, the definition of INR has been only based on the increase of aCD4 above different thresholds over a given time period. By using the CD4/CD8 ratio instead of aCD4, an even larger population would be defined with very few possibilities of IR based on CD4/CD8. However, this definition has to be validated in larger cohorts with a long follow-up time and correlated it with clinical events given the low concordance between both populations.

To date, the only available data on the aCD4 and CD4% variability are from a study by Gordon et al. conducted in 88 HIV-infected patients with suppressed viral loads for at least three years [7]. In this study, a median CV of 16.6 (IQR, 13.8–20.1) for aCD4, and 9.6 (IQR, 7.4–13.0) for CD4% were observed, which are comparable with our results. To the best of our knowledge, this is the first study that examines the CD4/CD8 variability in stable, virologically-suppressed subjects on ART. Our results show that the intra-annual variability of CD4/CD8 is one third lower than that of aCD4, and a 50% lower variability was observed for CD4% compared with that of aCD4. This lower variability should favor their use as markers of IR in patients receiving ART.

Regarding the CD8^+^ T cell dynamics, in untreated HIV infection, there is a disproportionate expansion of terminally differentiated CD8^+^ T cells, which leads to abnormal increases in lymphoid tissues and circulating aCD8. As described previously [34,35], during the first year on ART a decrease of aCD8 was observed, but only in the upper CD4 strata. By contrast, aCD8 increases proportionally to the aCD4 gain in the aCD4 stratum of ≤200/μl which would reflect a CD8^+^ T cell compartment regeneration after the HIV infection has become severe enough to result in profound lymphopenia.

Among the limitations of this study is the lack of information about the cytomegalovirus serologic status that has been shown to negatively impact CD4/CD8 restoration in the setting of effective ART [10,32,36,37].

In conclusion, our results suggest that both aCD4 and their increases after starting ART poorly correlate with CD4% and CD4/CD8 increments, and significantly overstate the IR rates assessed by CD4% and CD4CD8. Moreover, both CD4% and CD4/CD8 are more stable markers than aCD4 and should be taken into account to assess the IR rates in HIV-infected patients.

## Supporting information

S1 Fig Flowchart's study. ART, antiretroviral therapy; VL, viral load.
(PDF)

S1 Table Correlations between aCD4^+^, CD4^+^% and CD4^+^/CD8^+^ ratios. P-value <0.001 for all correlations.
(PDF)

S2 Fig Correlations between absolute CD4^+^ counts, CD4^+^ percentages and CD4^+^/CD8^+^ ratios. (PDF)

S3 Fig The increment of absolute CD4+ T cell counts, CD4 percentages and CD4/CD8 ratios according to years of follow up. A) Absolute and B) per cent changes in absolute CD4^+^ T cell counts, CD4^+^ percentages and CD4^+^/CD8^+^ ratios.
(PDF)

S4 Fig Dynamics of CD8^+^ T cell counts after starting antiretroviral therapy, according to CD4^+^ T cell strata. (PDF)

S2 Table Factors associated with the probability to achieve an absolute CD4+ T cell count ≥650/μl. Multivariable model I considered absolute CD4+ and CD8+ T cell counts, model II considered percentage of CD4+ and absolute CD8+ T cell counts, and model III consider CD4+/CD8+ ratio. HR, hazard ratio. CI95, confidence interval 95%.
(PDF)

S3 Table Factors associated with the probability to achieve a CD4+/CD8+ ratio ≥ 1. Multivariable model I considered absolute CD4+ (aCD4+) and CD8+ T cell counts, model II considered percentage of CD4+ and absolute CD8+ T cell counts, and model III consider CD4+/CD8+ ratio. HR, hazard ratio. CI95, confidence interval 95%.
(PDF)

S4 Table Factors associated with the probability to achieve an absolute CD4+ T cell count >650 plus a CD4+/CD8+ ratio ≥1 (Extensive Immune Recovery). Multivariable model I considered absolute CD4+ and CD8+ T cell counts, model II considered percentage of CD4+ and absolute CD8+ T cell counts, and model III consider CD4+/CD8+ ratio. HR, hazard ratio. CI95, confidence interval 95%.
(PDF)

S5 Table Characteristics of immunological non-responder patients. Characteristics of immunological non-responder patients based on absolute increment of CD4+ T cell counts (aCD4) ≤100 and ≤150 cells/μl after one and two years on treatment respectively, and of CD4/CD8 ratios (CD4/CD8) ≤0.1 and ≤0.15 after one and two years, respectively.
(PDF)
