# Supplementary figures and images for: Absolute CD4+ T cell count overstate immune recovery assessed by CD4+/CD8+ ratio in HIV-infected patients on treatment
Source: PLoS One. 2018 Oct 22;13(10):e0205777. doi: 10.1371/journal.pone.0205777 (PMC6197681; doi:10.1371/journal.pone.0205777)

**S1 Fig. Flowchart's study.**

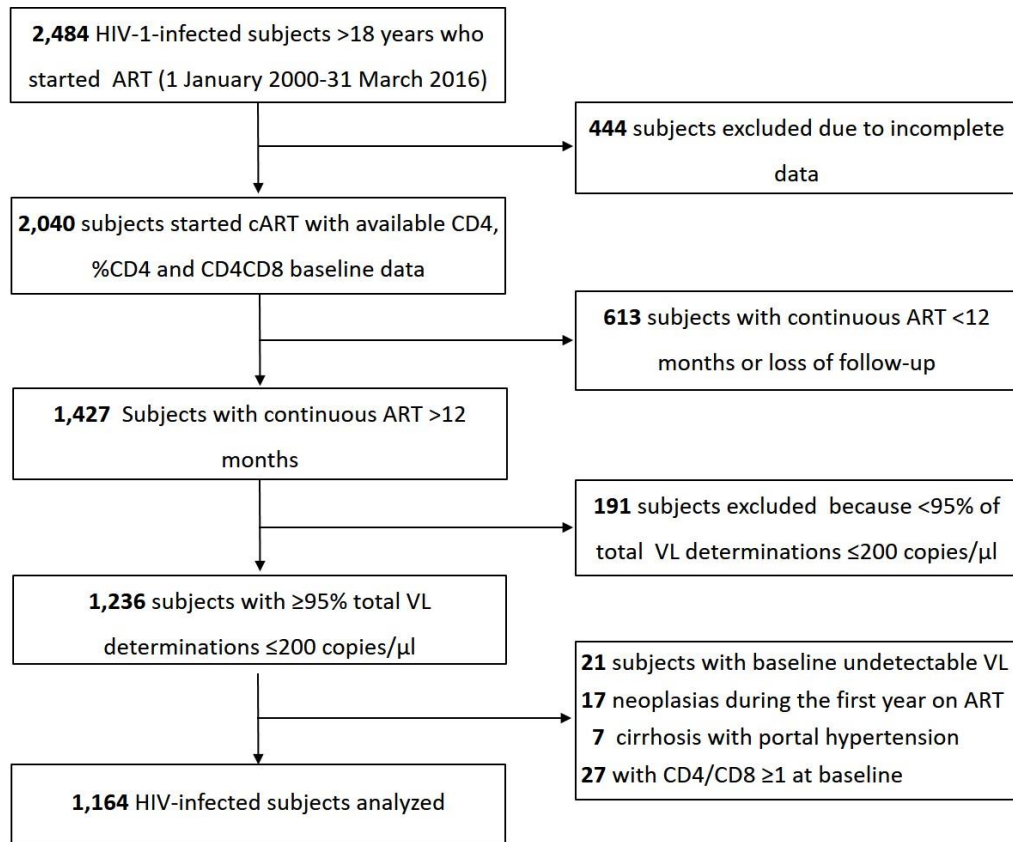

ART, antiretroviral therapy; VL, viral load.

Supplement: S1 Fig — ART, antiretroviral therapy; VL, viral load. (PDF) [file pone.0205777.s001.pdf]

**S2 Fig. Correlations between absolute CD4 counts, CD4 percentages and CD4/CD8 ratios.**

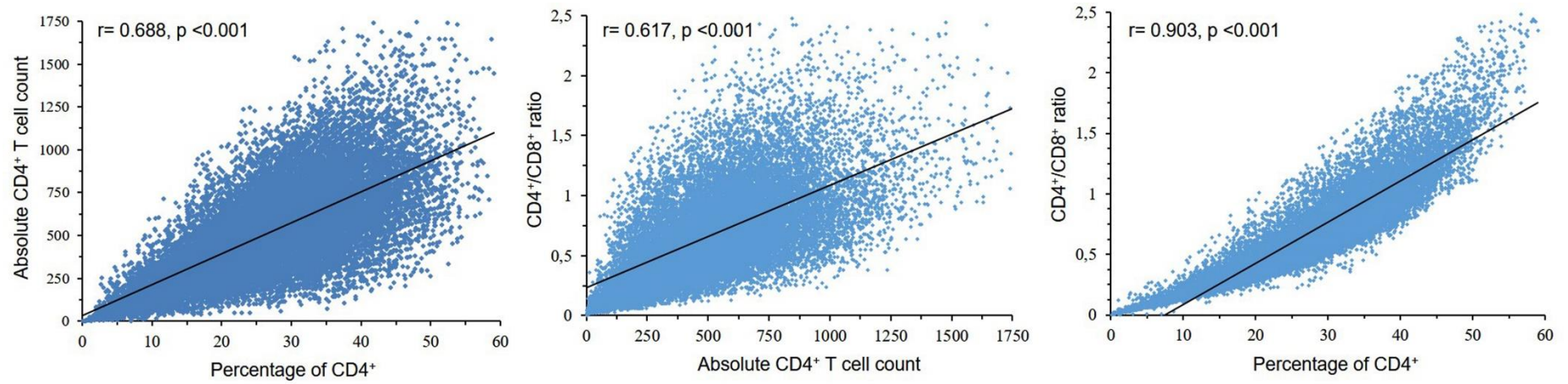

Supplement: S2 Fig — (PDF) [file pone.0205777.s003.pdf]
