# Supplementary material for: Absolute CD4+ T cell count overstate immune recovery assessed by CD4+/CD8+ ratio in HIV-infected patients on treatment
Source: PLoS One. 2018 Oct 22;13(10):e0205777. doi: 10.1371/journal.pone.0205777 (PMC6197681; doi:10.1371/journal.pone.0205777)
Supplement: S1 Table — P-value <0.001 for all correlations. (PDF) [file pone.0205777.s002.pdf]

S1 Table. Correlations between aCD4, CD4% and CD4/CD8 ratios.

		aCD4$^+$	CD4$^+$ %
≤200 CD4/µl (n= 2251)	aCD4$^+$	–	0.527
	CD4$^+$/CD8$^+$ ratio	0.431	0.917
201-350 CD4/µl (n= 4041)	aCD4$^+$	–	0.173
	CD4$^+$/CD8$^+$ ratio	0.223	0.890
351-500 CD4/µl (n= 4613)	aCD4$^+$	–	0.199
	CD4$^+$/CD8$^+$ ratio	0.167	0.864
501-650 CD4/µl (n= 4484)	aCD4$^+$	–	0.158
	CD4$^+$/CD8$^+$ ratio	0.136	0.851
>650 CD4/µl (n= 6485)	aCD4$^+$	–	0.334
	CD4$^+$/CD8$^+$ ratio	0.317	0.859

P-value <0.001 for all correlations.
