# Supplementary material for: Absolute CD4+ T cell count overstate immune recovery assessed by CD4+/CD8+ ratio in HIV-infected patients on treatment
Source: PLoS One. 2018 Oct 22;13(10):e0205777. doi: 10.1371/journal.pone.0205777 (PMC6197681; doi:10.1371/journal.pone.0205777)
Supplement: S3 Fig — A) Absolute and B) per cent changes in absolute CD4+ T cell counts, CD4+ percentages and CD4+/CD8+ ratios. (PDF) [file pone.0205777.s004.pdf]

S3 Fig. The increment of absolute CD4$^+$ T cell counts, CD4 percentages and CD4/CD8 ratios according to years of follow up.

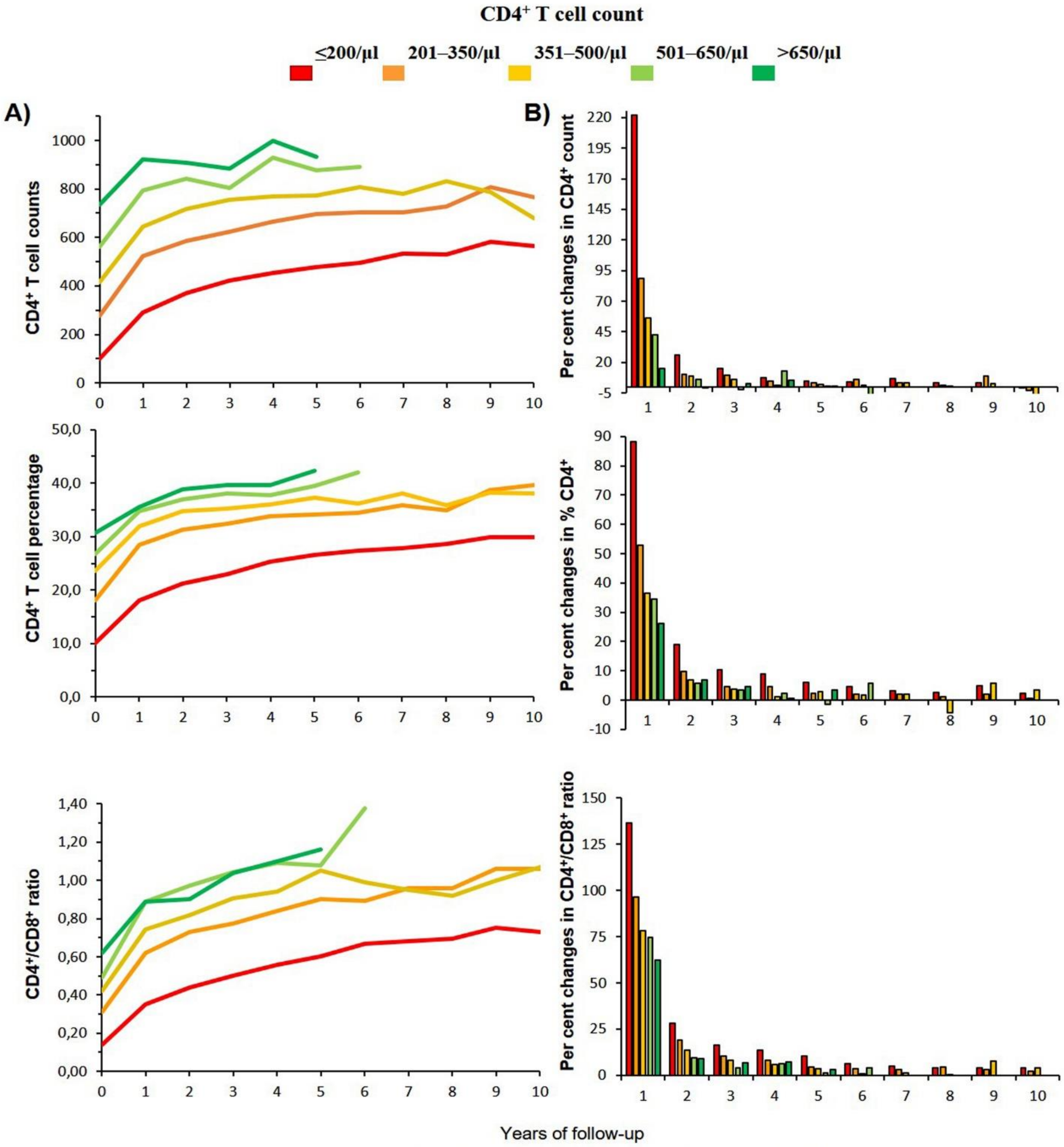

n	0	1	2	3	4	5	6	7	8	9	10
<=200	383	374	339	308	282	247	205	169	154	115	108
201-350	383	375	343	306	264	226	181	131	111	63	54
351-500	241	238	218	178	145	102	64	35	26	13	11
501-650	98	97	78	54	33	27	18				
>650	75	75	54	37	19	14					

A) Absolute and B) per cent changes in absolute CD4$^+$ T cell counts, CD4 percentages and CD4/CD8 ratios.
