# Supplementary material for: Absolute CD4+ T cell count overstate immune recovery assessed by CD4+/CD8+ ratio in HIV-infected patients on treatment
Source: PLoS One. 2018 Oct 22;13(10):e0205777. doi: 10.1371/journal.pone.0205777 (PMC6197681; doi:10.1371/journal.pone.0205777)
Supplement: S4 Fig — (PDF) [file pone.0205777.s005.pdf]

S4 Fig. Dynamics of CD8$^+$ T cell counts after starting antiretroviral therapy, according to CD4$^+$ T cell strata.

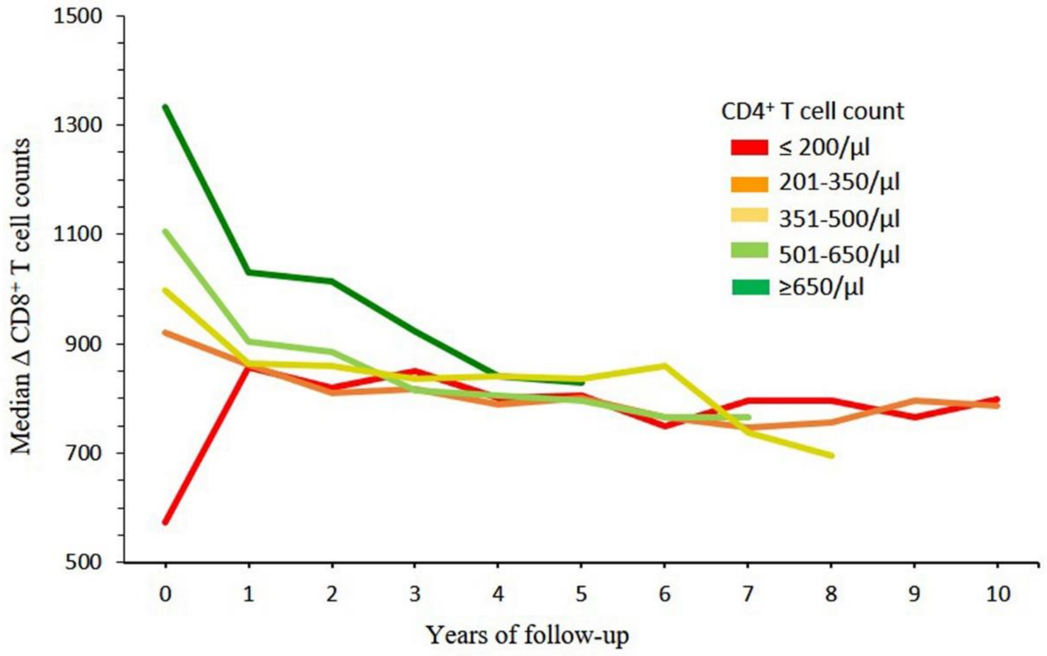

	Number of patients										
<=200	383	374	339	308	282	247	205	169	154	115	108
201-350	383	375	343	306	264	226	181	131	111	63	54
351-500	241	238	218	178	145	102	64	35	26		
501-650	98	97	78	54	33	27	18				
>650	75	75	54	37	19	14					
