# Supplementary material for: Absolute CD4+ T cell count overstate immune recovery assessed by CD4+/CD8+ ratio in HIV-infected patients on treatment
Source: PLoS One. 2018 Oct 22;13(10):e0205777. doi: 10.1371/journal.pone.0205777 (PMC6197681; doi:10.1371/journal.pone.0205777)
Supplement: S2 Table — Multivariable model I considered absolute CD4+ and CD8+ T cell counts, model II considered percentage of CD4+ and absolute CD8+ T cell counts, and model III consider CD4+/CD8+ ratio. HR, hazard ratio. CI95, confidence interval 95%. (PDF) [file pone.0205777.s006.pdf]

S2 Table. Factors associated with the probability to achieve an absolute CD4+ T cell count ≥650/µl.

Variables	Univariate		Multivariate I		Multivariate II		Multivariate II	
	HR (IC_{95})	*p*	HR (IC_{95})	*p*	HR (IC_{95})	*p*	HR (IC_{95})	*p*
Age (per 10 years more)	0.87 (0.80–0.94)	<0.001	0.98 (0.91–1.06)	0.594	0.96 (0.89–1.04)	0.309	0.92 (0.74–1.10)	0.228
HIV RNA (per log_{10} higher)	0.87 (0.79–0.95)	0.004	1.19 (1.07–1.33)	0.001	1.14 (1.02–1.27)	0.018	1.05 (0.94–1.17)	0.370
Woman (vs. male)	0.89 (0.72−1.10)	0.270						
HCV-ARN positive (vs. negative)	0.52 (0.42–0.66)	<0.001	0.74 (0.58−0.94)	0.015	0.77 (0.61–0.98)	0.032	0.70 (0.55–0.89)	0.004
HBV Ag positive (vs. negative)	0.94 (0.65−1.36)	0.748						
$CD8^+$ T-cell count	1.74 (1.54−1.98)	<0.001	0.99 (0.85−1.15)	0.907	2.03 (1.76−2.35)	0.001		
Absolute $CD4^+$ T-cell count								
≤200	0.33 (0.27–0.41)	<0.001	0.31 (0.25–0.40)	<0.001				
201–350	(ref.)		(ref.)					
351–500	1.89 (1.57–2.27)	<0.001	1.84 (1.52–2.23)	<0.001				
>500	3.48 (2.82–.31)	<0.001	3.31 (2.64-4.15)	<0.001				
CD4 percentage								
≤16%	0.43 (0.36–0.52)	<0.001			0.44 (0.36–0.53)	<0.001		
16.1–24%	(ref.)				(ref.)			
24.1–32%	1.44 (1.19–1.73)	<0.001			1.55 (1.28–1.87)	<0.001		
>32	1.73 (1.33–2.25)	<0.001			2.12 (1.62–2.78)	<0.001		
CD4/CD8 ratio								
<0.30	0.47 (0.40–0.56)	<0.001					0.52 (0.43–0.62)	<0.001
0.30–0.50	(ref.)						(ref.)	
0.51--0.79	1.46 (1.20–1.76)	<0.001					1.39 (1.15–1.69)	<0.001
≥0.8	1.79 (1.21–2.67)	<0.001					1.76 (1.18–2.62)	<0.001
Period of ART introduction								
2000-2005	(ref.)		(ref.)		(ref.)		(ref.)	
2006_2010	1.49 (1.20–1.84)	<0.001	0.95 (0.75–1.19)	0.652	1.04 (0.82–1.30)	0.769	1.19 (0.95–1.49)	0.124
≥2011	2.56 (2.06–3.19)	<0.001	1.20 (0.94–1.52)	0.146	1.35 (1.06–1.72)	0.014	1.80 (1.42–2.27)	<0.001

Multivariable model I considered absolute $CD4^+$ and $CD8^+$ T cell counts, model II considered percentage of $CD4^+$ and absolute $CD8^+$ T cell counts, and model III consider CD4/CD8 ratio. HR, hazard ratio. CI_{95}, confidence interval 95%.
