# Supplementary material for: Absolute CD4+ T cell count overstate immune recovery assessed by CD4+/CD8+ ratio in HIV-infected patients on treatment
Source: PLoS One. 2018 Oct 22;13(10):e0205777. doi: 10.1371/journal.pone.0205777 (PMC6197681; doi:10.1371/journal.pone.0205777)
Supplement: S3 Table — Multivariable model I considered absolute CD4+ (aCD4+) and CD8+ T cell counts, model II considered percentage of CD4+ and absolute CD8+ T cell counts, and model III consider CD4+/CD8+ ratio. HR, hazard ratio. CI95, confidence interval 95%. (PDF) [file pone.0205777.s007.pdf]

S3 Table. Factors associated with the probability to achieve a CD4+/CD8+ ratio ≥ 1.

	Univariate		Multivariate I		Multivariate II		Multivariate II	
**Variables**	**HR (IC_{95})**	***p***	**HR (IC_{95})**	***p***	**HR (IC_{95})**	***p***	**HR (IC_{95})**	***p***
Age (per 10 years more)	0.84 (0.75–0.92)	<0.001	0.97 (0.88–1.07)	0.590	0.93 (0.85–1.03)	0.145	0.92 (0.84–1.02)	0.126
HIV RNA (per log_{10} higher)	0.79 (0.70–0.90)	<0.001	1.09 (0.95–1.25)	0.240	1.19 (0.95–1.27)	0.192	1.15 (0.99–1.32)	0.058
Woman (vs. male)	1.61 (1.28–2.02)	<0.001	1.68 (1.31–2.14)	<0.001	1.50 (1.18–1.91)	0.001	1.62 (1.28–2.04)	<0.001
HCV-ARN positive (vs. negative)	0.53 (0.41–0.73)	<0.001	0.70 (0.50–0.94)	0.019	0.69 (0.51–0.94)	0.018	0.76 (0.56–1.03)	0.075
HBV Ag positive (vs. negative)	0.92 (0.58–1.46)	0.730						
$CD8^+$ T-cell count	0.88 (0.77–1.00)	0.057	0.40 (0.33–0.48)	<0.001	0.89 (0.75–1.05)	0.163		
Absolute $CD4^+$ T-cell count								
≤200	0.40 (1.15–1.83)	0.002	0.19 (0.13–0.26)	0.001				
201–350	(ref.)		(ref.)					
351–500	2.19 (1.75–2.74)	<0.001	1.60 (1.26–2.04)	<0.001				
>500	3.65 (2.71–4.91)	<0.001	3.07 (2.32–4.07)	<0.001				
CD4 percentage								
≤16%	0.34 (0.26–0.43)	<0.001			0.33 (0.25–0.43)	<0.001		
16.1–24%	(ref.)				(ref.)			
24.1–32%	1.45 (1.15–1.83)	<0.001			2.05 (1.63–2.58)	<0.001		
>32	2.37 (1.83–3.08)	<0.001			3.27 (2.41–4.45)	<0.001		
CD4/CD8 ratio								
<0.30	0.31 (0.25–0.39)	<0.001					0.31 (0.24–0.40)	<0.001
0.30–0.50	(ref.)						(ref.)	
0.51--0.79	2.40 (1.92–2.99)	<0.001					2.33 (1.86–2.91)	<0.001
≥0.8	5.24 (3.51–7.81)	<0.001					5.33 (3.56–7.97)	<0.001
Period of ART introduction								
2000-2005	(ref.)		(ref.)		(ref.)		(ref.)	
2006_2010	1.39 (1.06–1.81)	0.017	0.95 (0.72–1.26)	0.722	0.95 (0.72–1.26)	0.728	1.00 (0.76–1.32)	1.000
≥2011	2.14 (1.62–2.82)	<0.001	1.18 (0.87–1.60)	0.484	1.09 (0.81–1.47)	0.584	1.21 (0.90–1.63)	0.203

Multivariable model I considered absolute CD4 (aCD4) and $CD8^+$ T cell counts, model II considered percentage of $CD4^+$ and absolute $CD8^+$ T cell counts, and model III consider CD4/CD8 ratio. HR, hazard ratio. CI_{95}, confidence interval 95%.
