# Supplementary material for: Absolute CD4+ T cell count overstate immune recovery assessed by CD4+/CD8+ ratio in HIV-infected patients on treatment
Source: PLoS One. 2018 Oct 22;13(10):e0205777. doi: 10.1371/journal.pone.0205777 (PMC6197681; doi:10.1371/journal.pone.0205777)
Supplement: S4 Table — Multivariable model I considered absolute CD4+ and CD8+ T cell counts, model II considered percentage of CD4+ and absolute CD8+ T cell counts, and model III consider CD4+/CD8+ ratio. HR, hazard ratio. CI95, confidence interval 95%. (PDF) [file pone.0205777.s008.pdf]

S4 Table. Factors associated with the probability to achieve an absolute CD4+ T cell count >650 plus a CD4/CD8 ratio ≥1 (Extensive Immune Recovery).

	Univariate		Multivariate I		Multivariate II		Multivariate II	
**Variables**	**HR (IC_{95})**	***p***	**HR (IC_{95})**	***p***	**HR (IC_{95})**	***p***	**HR (IC_{95})**	***p***
Age (per 10 years more)	0.82 (0.7-–0.92)	<0.001	0.97 (0.86–1.09)	0.586	0.94 (0.84–1.06)	0.320	0.92 (0.82–1.04)	0.174
HIV RNA (per log_{10} higher)	0.79 (0.68–0.90)	0.001	1.19 (1.01–1.41)	0.035	1.19 (1.01–1.40)	0.044	1.20 (1.01–1.41)	0.035
Woman (vs. male)	1.37 (1.05–1.80)	0.021	1.54 (1.5–2.06)	0.004	1.37 (1.03–1.82)	0.028	1.40 (1.06–1.85)	0.017
HCV-ARN positive (vs. negative)	0.38 (0.26–0.55)	<0.001	0.55 (0.37–0.82)	0.003	0.57 (0.38–0.85)	0.004	0.56 (0.38–0.83)	0.004
HBV Ag positive (vs. negative)	0.95 (0.57–1.68)	0.839						
$CD8^+$ T-cell count	1.03 (0.88–1.21)	0.680	0.38 (0.31–0.48)	<0.001	1.15 (0.94–1.41)	0.176		
Absolute $CD4^+$ T-cell count								
≤200	0.35 (0.25–0.48)		0.15 (0.10–0.23)	<0.001				
201–350	(ref.)	<0.001	(ref.)					
351–500	1.93 (1.47–2.52)	<0.001	2.14 (1.62–2.83)	<0.001				
>500	4.09 (3.05–5.46)	<0.001	5.36 (3.91–7.36)	<0.001				
$CD4^+$ percentage								
≤16%	0.31 (0.23–0.43)	<0.001			0.32 (0.23–0.44)	<0.001		
16.1–24%	(ref.)				(ref.)			
24.1–32%	2.71 (2.10–3.50)	<0.001			2.60 (2.00–3.37)	<0.001		
>32	4.30 (3.10–5.95)	<0.001			4.25 (3.02–5.98)	<0.001		
CD4/CD8 T -cell ratio								
<0.30	0.29 (0.21–0.38)						0.29 (0.22–0.40)	<0.001
0.30–0.50	(ref.)	<0.001					(ref.)	
0.51--0.79	2.58 (2.01–3.30)	<0.001					2.49 (1.93–3.20)	<0.001
≥0.8	5.97 (3.87–9.19)	<0.001					5.36 (3.23-8.63)	<0.001
Period of ART introduction								
2000-2005	(ref.)		(ref.)		(ref.)		(ref.)	
2006_2010	1.57 (1.14–2.17)	0.006	0.99 (0.71–1.44)	0.986	1.01 (0.72–1.41)	0.956	1.02 (0.77–1.51)	0.647
≥2011	3.01 (2.15–4.21)	<0.001	1.34 (0.93–1.94)	0.119	1.31 (0.91–1.89)	0.143	1.58 (1.10–2.25)	0.012

Multivariable model I considered absolute CD4 (aCD4) and $CD8^+$ T cell counts, model II considered percentage of $CD4^+$ and absolute $CD8^+$ T cell counts, and model III consider CD4/CD8 ratio. HR, hazard ratio. CI_{95}, confidence interval 95%.
