# Supplementary material for: Absolute CD4+ T cell count overstate immune recovery assessed by CD4+/CD8+ ratio in HIV-infected patients on treatment
Source: PLoS One. 2018 Oct 22;13(10):e0205777. doi: 10.1371/journal.pone.0205777 (PMC6197681; doi:10.1371/journal.pone.0205777)
Supplement: S5 Table — Characteristics of immunological non-responder patients based on absolute increment of CD4+ T cell counts (aCD4) ≤100 and ≤150 cells/μl after one and two years on treatment respectively, and of CD4/CD8 ratios (CD4/CD8) ≤0.1 and ≤0.15 after one and two years, respectively. (PDF) [file pone.0205777.s009.pdf]

S5 Table. Characteristics of immunological non-responder patients.

	**Δ aCD4 ≤ 100/150** (n= 80)	**Δ CD4/CD8 ≤ 0.10/0.15** (n= 168)
**Male sex**, no. (%)	68 (85)	156 (92.9)
**Age** (years)	41 (35–47) [22–73]	39 (33–47) [22–73]
**HIV-RNA**, log_{10} copies/ml	4.64 (3.92–5.14) [2.04–5.85]	4.85 (4.39–5.30) [2.04–6.62]
**Absolute CD4/µl**	163 (66–294) [3–389]	157 (64–337) [2–765]
**Percentage of CD4+**	13.9 (6.2–18.4) [1.0–27.8]	13.5 (6.7–21.9) [0.1–36.5]
**CD4/CD8 ratio**	0.21 (0.09–0.31) [0.01–0.54]	0.22 (0.10–0.41) [0.01–0.70]
**HCV-RNA +**, no. (%)	30 (37.5)	46 (27.4)
**AIDS events**, no. (%)	25 (31.3)	54 (32.1)
**Probability of aCD4 ≥650** after 5 years	2.2% (CI_{95}, 0.0–6.5)	38.5% (CI_{95}, 30.3–47.0)
**Probability of CD4/CD8 ≥1** after 5 years	13.8% (CI_{95}, 4.8–23.8)	6.1% (CI_{95}, 1.6–10.6)
**Probability of aCD4 ≥650 + CD4/CD8 ≥1** after 5 years	0%	2.5% (CI_{95}, 0.0–5.0)

Characteristics of immunological non-responder patients based on absolute increment of $CD4^+$ T cell counts (aCD4) ≤100 and ≤150 cells/µl after one and two years on treatment respectively, and of CD4/CD8 ratios (CD4/CD8) ≤0.1 and ≤0.15 after one and two years, respectively.
